# Dynamic regulation of dynein localization revealed by small molecule inhibitors of ubiquitination enzymes

**DOI:** 10.1098/rsob.180095

**Published:** 2018-09-26

**Authors:** Julie K. Monda, Iain M. Cheeseman

**Affiliations:** 1 Whitehead Institute for Biomedical Research, 455 Main Street, Cambridge, MA 02142, USA; 2 Department of Biology, Massachusetts Institute of Technology, Cambridge, MA 02142, USA

**Keywords:** dynein, ubiquitin, NSC697923, PYR-41, mitosis, microtubules

## Abstract

Cytoplasmic dynein is a minus-end-directed microtubule-based motor that acts at diverse subcellular sites. During mitosis, dynein localizes simultaneously to the mitotic spindle, spindle poles, kinetochores and the cell cortex. However, it is unclear what controls the relative targeting of dynein to these locations. As dynein is heavily post-translationally modified, we sought to test a role for these modifications in regulating dynein localization. We find that dynein rapidly and strongly accumulates at mitotic spindle poles following treatment with NSC697923, a small molecule that inhibits the ubiquitin E2 enzyme, Ubc13, or treatment with PYR-41, a ubiquitin E1 inhibitor. Subsets of dynein regulators such as Lis1, ZW10 and Spindly accumulate at the spindle poles, whereas others do not, suggesting that NSC697923 differentially affects specific dynein populations. We additionally find that dynein relocalization induced by NSC697923 or PYR-41 can be suppressed by simultaneous treatment with the non-selective deubiquitinase inhibitor, PR-619. However, we did not observe altered dynein localization following treatment with the selective E1 inhibitor, TAK-243. Although it is possible that off-target effects of NSC697923 and PYR-41 are responsible for the observed changes in dynein localization, the rapid relocalization upon drug treatment highlights the highly dynamic nature of dynein regulation during mitosis.

## 1. Introduction

Dynein is a AAA+ ATPase that uses the energy from ATP hydrolysis to walk along a microtubule [1]. Dynein localizes to diverse sites within the cell, including the mitotic spindle, the centrosomes, the nuclear envelope, the kinetochores and the cell cortex, where it performs numerous roles in interphase and mitosis [2]. This subcellular targeting is accomplished in part by the association of the dynein complex with various regulatory and adaptor proteins, including the dynactin complex, NuMA, Spindly, Bicaudal D2, Nde1 (also known as NudE) and others [3].

Dynein is also heavily post-translationally modified [4], suggesting the presence of additional mechanisms for regulating its localization and function. For example, multiple sites within dynein are modified by ubiquitin [4–11]. The most well-characterized function of ubiquitination is to target a substrate for degradation by the 26S proteasome [12], but ubiquitination also plays non-degradative roles in regulating diverse processes such as endocytosis and DNA repair [13,14]. Additionally, protein–protein interactions and localization can be regulated by ubiquitination [15]. Prior work has identified several ubiquitination sites within the dynein complex or dynein regulatory proteins that do not increase in abundance upon proteasome inhibition [6], and therefore may alter dynein function through non-degradative mechanisms. Indeed, evidence suggests that the dynein–NuMA interaction is enhanced by non-degradative ubiquitination [16], and a role for non-degradative ubiquitination has also been implicated in indirectly targeting dynein to the cell cortex [17].

Ubiquitination is mediated by the sequential actions of an E1 activating enzyme, an E2 conjugating enzyme and an E3 ligase. Polyubiquitin chains can be built from any of seven internal lysines or the N-terminal methionine of ubiquitin [12]. The type of linkage used in building the polyubiquitin chain helps to determine the downstream effect on the ubiquitinated substrate [12]. For example, proteasome targeting is typically directed by the synthesis of a lysine 48-linked polyubiquitin chain [12], whereas a K63-linked chain is the most abundant non-degradative ubiquitination event [18]. The E2 enzymes typically specify the type of linkage generated, with Ubc13 acting as the primary ubiquitin E2 enzyme involved in K63-linked non-degradative chain formation [13]. However, the cellular processes regulated by non-degradative ubiquitination remain incompletely understood.

Here, we use a small molecule inhibitor of Ubc13, as well as inhibitors targeting other components of the ubiquitin conjugation machinery to reveal the highly dynamic nature of dynein localization in mitotic cells.

## 2. Results

### 2.1. Dynein rapidly accumulates at the spindle poles following Ubc13 inhibition by NSC697923 treatment

**Figure 1.**
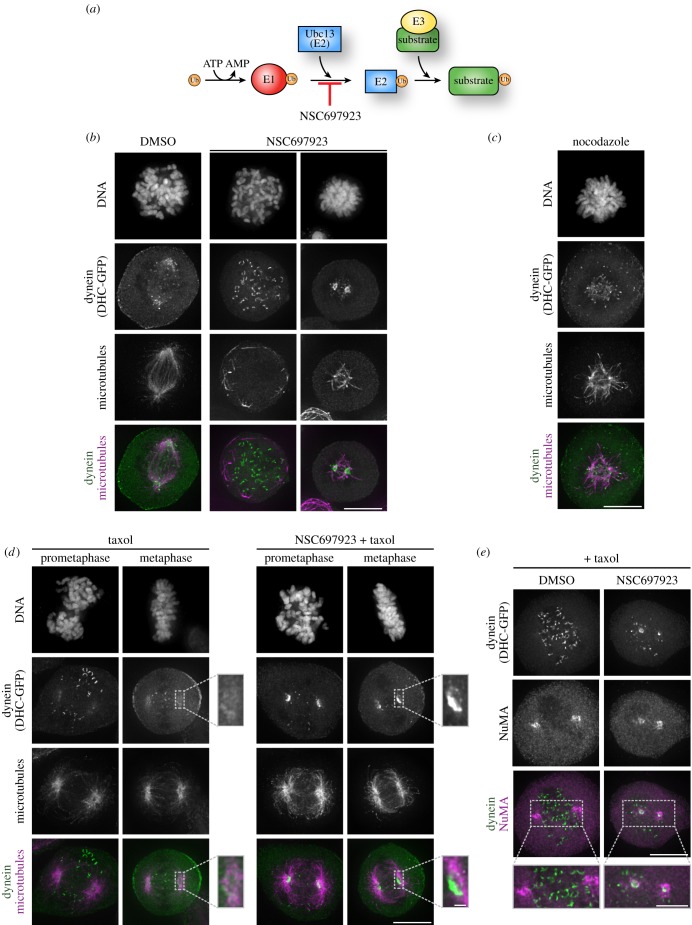
NSC697923 induces accumulation of dynein at the mitotic spindle poles. (*a*) Cartoon of the ubiquitin conjugation pathway. NSC697923 is a small molecule inhibitor of the ubiquitin E2 enzyme, Ubc13. (*b–d*) Immunofluorescence images of DNA (Hoechst), stably expressed dynein heavy chain-GFP (DHC-GFP), and microtubules (DM1A) after treating the cells for 15 min with the indicated compounds. Scale bars, 10 µm; 1 µm in the magnified panel. (*e*) Representative immunofluorescence images of stably expressed dynein heavy chain-GFP (DHC-GFP) and NuMA after treatment for 15 min with NSC697923 and taxol. Scale bar, 10 µm; 5 µm in the magnified panel.

We hypothesized that post-translational modifications may play a critical role in controlling the subcellular localization of the cytoplasmic dynein complex. We therefore sought to identify perturbations that alter dynein localization. To begin, we treated HeLa cells stably expressing GFP-tagged dynein heavy chain (DHC-GFP) with inhibitors of mitotic kinases and phosphatases to perturb cellular phosphorylation. We did not observe any obvious changes in the mitotic localization of dynein following transient inhibition of Aurora kinases (ZM447439/VX680), cyclin-dependent kinase (flavopiridol), Mps1 (AZ3146), Plk1 (BI2536), PP1 or PP2A (okadaic acid) (data not shown). We next tested for a contribution from ubiquitination to dynein localization by treating with the Ubc13 inhibitor, NSC697923 (figure 1*a*) [19]. After only 15 min of treatment, NSC697923 caused a nearly complete disassembly of the mitotic spindle in 23 of 100 mitotic cells analysed (figure 1*b*). Strikingly, in the majority of cells with spindle microtubules remaining, we observed a dramatic accumulation of dynein at two foci that correspond to the spindle poles (figure 1*b*). Treatment with a low dose of nocodazole resulted in a similar disruption of the mitotic spindle, but did not induce dynein accumulation at the spindle poles, suggesting that the NSC697923-induced effects on dynein localization are independent of its effects on the mitotic spindle (figure 1*c*). To further test this, we stabilized the mitotic spindle by treating cells with the microtubule-stabilizing drug taxol. Taxol treatment alone did not alter the localization of dynein (figure 1*d*). We next simultaneously treated cells with NSC697923 and taxol. Under these conditions, the mitotic spindle was largely preserved and we again observed strong dynein relocalization to the spindle poles in 79 ± 6% of cells in either prometaphase or metaphase, compared with only 1 ± 2% of cells treated with taxol alone (figure 1*d*; see also figure 6). Line scan analyses confirmed an increase in dynein intensity at the spindle poles following NSC697923 treatment (electronic supplementary material, figure S1A). This suggests that the change in dynein localization is not a secondary consequence of disrupting the structure of the mitotic spindle. Co-staining for NuMA, one of the outermost proteins at the spindle pole [20], revealed that NuMA localization is unaffected by NSC697923 treatment (figure 1*e*; electronic supplementary material, figure S1B) and that the spindle pole-localized dynein was slightly internal to the localization of NuMA (figure 1*e*). Together, our data demonstrate a rapid accumulation of dynein at the spindle poles following the addition of the small molecule inhibitor, NSC697923.

### 2.2. A subset of dynein-associated factors accumulate at spindle poles after NSC697923 treatment

**Figure 2.**
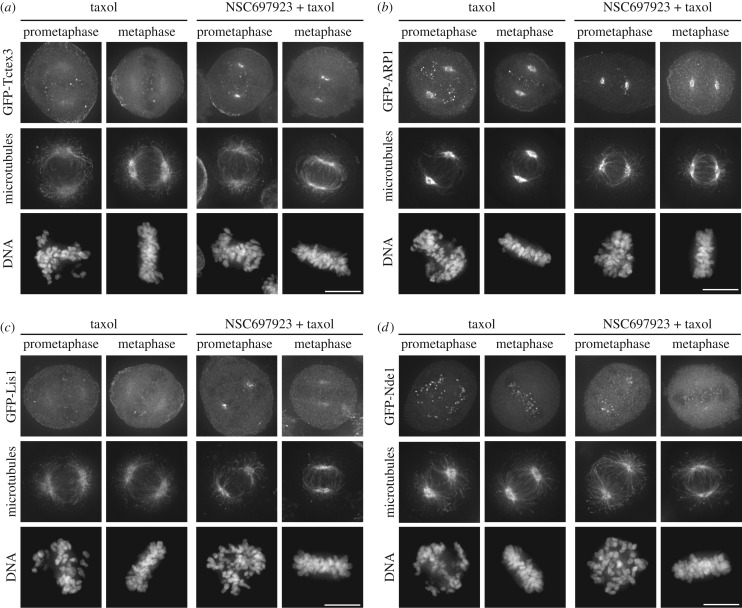
NSC697923 induces accumulation of a subset of dynein regulators at the mitotic spindle poles. (*a*) Representative immunofluorescence images of stably expressed GFP-Tctex-type 3, microtubules (DM1A) and DNA (Hoechst) after treating the cells for 15 min with the indicated compounds. Scale bar, 10 µm. (*b*) Representative immunofluorescence images of stably expressed GFP-ARP1, microtubules (DM1A), and DNA (Hoechst) after treating the cells for 15 min with the indicated compounds. Scale bar, 10 µm. (*c*) Representative immunofluorescence images of stably expressed GFP-Lis1, microtubules (DM1A) and DNA (Hoechst) after treating the cells for 15 min with the indicated compounds. Scale bar, 10 µm. (*d*) Representative immunofluorescence images of stably expressed GFP-Nde1, microtubules (DM1A) and DNA (Hoechst) after treating the cells for 15 min with the indicated compounds. Scale bar, 10 µm.

Dynein heavy chain associates with numerous proteins, including other subunits of the dynein complex, as well as various adaptor proteins [3]. We therefore sought to determine which other proteins accumulate with dynein heavy chain at the spindle poles after NSC697923 treatment. As expected, another subunit of the dynein complex, the dynein light chain Tctex-type 3, also accumulated at the mitotic spindle poles following NSC697923 addition (figure 2*a*). By contrast, ARP1, a subunit of the dynactin complex and a key regulator of dynein, strongly localizes to mitotic spindle poles in untreated cells, with no obvious alteration in localization following NSC697923 treatment (figure 2*b*). Next, we examined Lis1 and Nde1, proteins that coordinately regulate diverse functions of dynein [3,21]. Lis1 strongly accumulated at the spindle poles following NSC697923 treatment during prometaphase (figure 2*c*), whereas the localization of Nde1 was comparatively unaffected in both prometaphase and metaphase (figure 2*d*). Because only a subset of dynein-interacting proteins relocalize to the spindle poles after NSC697923 treatment, these data suggest that NSC697923 differentially affects specific dynein complexes.

**Figure 3.**
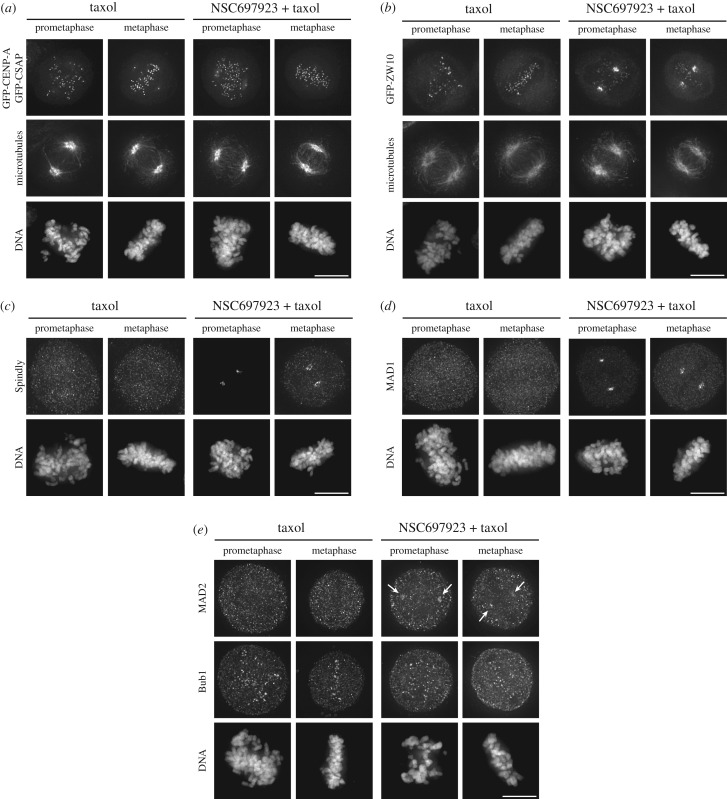
NSC697923 induces accumulation of a subset of dynein interactors at the mitotic spindle poles. (*a*) Representative immunofluorescence images of stably expressed GFP-CENP-A and GFP-CSAP, microtubules (DM1A) and DNA (Hoechst) after treating the cells for 15 min with the indicated compounds. Scale bar, 10 µm. (*b*) Representative immunofluorescence images of stably expressed GFP-ZW10, microtubules (DM1A) and DNA (Hoechst) after treating the cells for 15 min with the indicated compounds. Scale bar, 10 µm. (*c*) Representative immunofluorescence images of Spindly and DNA (Hoechst) after treating the cells for 15 min with the indicated compounds. Scale bar, 10 µm. (*d*) Representative immunofluorescence images of MAD1 and DNA (Hoechst) after treating the cells for 15 min with the indicated compounds. Scale bar, 10 µm. (*e*) Representative immunofluorescence images of MAD2, Bub1 and DNA (Hoechst) after treating the cells for 15 min with the indicated compounds. Arrows indicate weak, but detectable localization of MAD2 at the spindle poles. Scale bar, 10 µm.

We next sought to test the localization of diverse mitotic proteins following NSC697923 treatment. In addition to the spindle pole-localized protein NuMA (figure 1*e*), we found that the centriole and spindle-localized protein CSAP [22] appeared unaffected by treatment with NSC697923 (figure 3*a*). The centromere-localized protein, CENP-A, was similarly unaffected by NSC697923 treatment (figure 3*a*). Thus, NSC697923 does not globally disrupt mitotic protein function.

The Rod/Zwilch/ZW10 (RZZ) complex and Spindly are involved in targeting dynein to mitotic kinetochores [3] and have been proposed to be cargo of the dynein complex [23]. In agreement with this, we found that ZW10 and Spindly accumulated at the spindle poles after treatment with NSC697923 (figure 3*b*,*c*). The RZZ complex also plays a key role in targeting the spindle assembly checkpoint proteins, MAD1 and MAD2, to mitotic kinetochores [24–26]. We found that MAD1 clearly accumulates at the spindle poles following NSC697923 treatment (figure 3*d*). The behaviour of MAD2 was more variable, but we typically saw some localization of MAD2 at the spindle poles in NSC697923-treated cells that we did not observe in control cells (figure 3*e*). By contrast, the localization of Bub1, a key protein involved in targeting the RZZ complex to mitotic kinetochores [24,27], appeared unaffected by NSC697923 treatment (figure 3*e*). Together, these data define a subset of dynein-interacting proteins that show altered localization following treatment with NSC697923.

### 2.3. Proteasome inhibition does not alter dynein localization in mitosis

**Figure 4.**
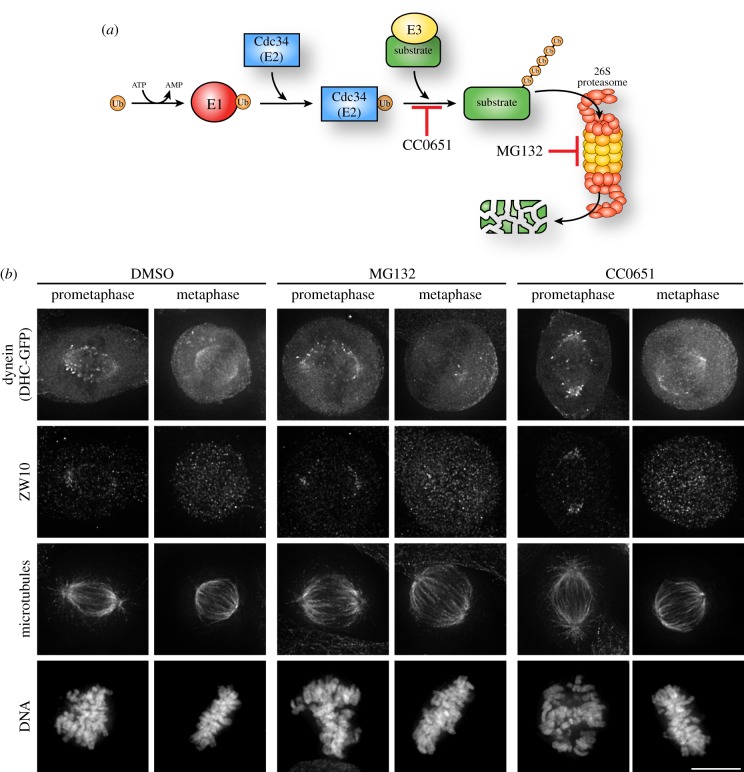
Dynein localization is not controlled by degradative ubiquitination. (*a*) Cartoon of the ubiquitin conjugation pathway illustrating the ubiquitin E2 enzyme, Cdc34, and the targeting of substrates for degradation by the 26S proteasome. CC0651 inhibits Cdc34 and MG132 inhibits the proteasome. (*b*) Representative immunofluorescence images of stably expressed dynein heavy chain-GFP (DHC-GFP), ZW10, microtubules (DM1A) and DNA (Hoechst) after treating the cells for 15 min with the indicated compounds. Scale bar, 10 µm.

Although K63-linked polyubiquitination is not a canonical signal for degradation, K63 chains can still alter substrate stability [12]. To test whether the relocalization of dynein following NSC697923 treatment was due to altered protein stability, we assessed dynein localization following inhibition of the proteasome with MG132 (figure 4*a*). In contrast to the relocalization induced by NSC697923 treatment, proteasome inhibition did not induce an accumulation of dynein at spindle poles (figure 4*b*). We also visualized endogenous ZW10 in these cells by immunofluorescence and did not observe signal that noticeably differed from DMSO-treated cells (figure 4*b*). Proteasomal targeting is frequently accomplished by K48-linked polyubiquitin chains, and Cdc34 is a key E2 enzyme involved in K48-linked polyubiquitin chain formation [28]. We therefore also used CC0651 [29], an inhibitor of Cdc34, and again did not observe a change in dynein or ZW10 localization (figure 4*b*). Collectively, these results suggest that the mitotic localization of dynein is not regulated by a degradative ubiquitination event.

### 2.4. Ubiquitin E1 inhibitors have differential effects on dynein localization in mitosis

**Figure 5.**
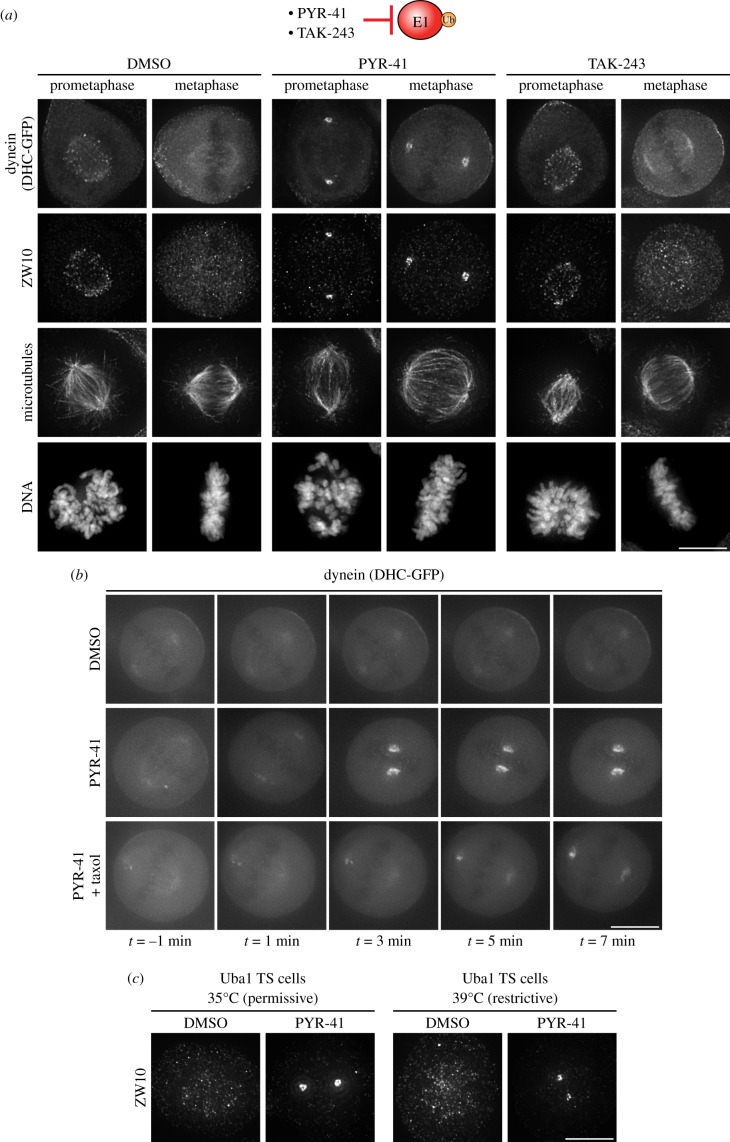
Dynein accumulation at the spindle poles is induced by PYR-41. (*a*) Top: Cartoon illustrating inhibition of the ubiquitin E1 enzyme by PYR-41 and TAK-243. Bottom: Representative immunofluorescence images of stably expressed dynein heavy chain-GFP (DHC-GFP), ZW10, microtubules (DM1A) and DNA (Hoechst) after treating the cells for 15 min with the indicated compounds. Scale bar, 10 µm. (*b*) Representative images of live cells stably expressing dynein heavy chain-GFP (DHC-GFP) before and after addition of the indicated drugs at *t* = 0. Scale bar, 10 µm. (*c*) Representative immunofluorescence images of ZW10 in TS20 cells after treating the cells for 15 min with the indicated compounds. Scale bar, 10 µm.

Ubiquitination requires the sequential activities of three distinct enzymes. The E2 enzymes, such as Ubc13, rely on an upstream ubiquitin activation step performed by a ubiquitin E1 enzyme. We therefore sought to further test whether the relocalization of dynein induced by NSC697923 treatment is due to inhibition of ubiquitination by treating cells with PYR-41 [30] or TAK-243 [31], small molecule inhibitors that target the E1 enzyme. As we observed for NSC697923 treatment, PYR-41 rapidly induced the accumulation of dynein and ZW10 at spindle poles (figure 5*a*). Indeed, by live-cell imaging, increased dynein levels were observed at poles within 5 min of addition of PYR-41 in both the absence and presence of taxol to stabilize the mitotic spindle (figure 5*b*). However, the recently developed E1 inhibitor, TAK-243, did not affect dynein localization (figure 5*a*). TAK-243 is a potent inhibitor of both Uba1 and the other human ubiquitin E1 enzyme, Uba6 [31]. Thus, the lack of dynein relocalization following TAK-243 treatment suggests that the NSC697923 and PYR-41-induced spindle pole accumulation of dynein could be due to off-target effects. To test this possibility, we used the TS20 cell line that contains temperature-sensitive mutations in Uba1 [32,33], the primary ubiquitin E1 enzyme. At the restrictive temperature, Uba1 levels should be significantly reduced, yet we did not observe an accumulation of ZW10 at the spindle poles (figure 5*c*). Additionally, in the absence of Uba1, the cells should be insensitive to any on-target effects of PYR-41. However, we found that PYR-41 still induced ZW10 accumulation at the spindle poles at the restrictive temperature (figure 5*c*). We were also unable to validate the specificity of PYR-41 using a CRISPR/Cas9-based system [34] to eliminate Uba1 from HeLa cells (data not shown). Therefore, it is possible that the relocalization of dynein induced by PYR-41 and NSC697923 is not due to directly interfering with ubiquitination, but instead is caused by off-target effects.

### 2.5. NSC697923- and PYR-41-induced dynein relocalization is suppressed by simultaneous treatment with the deubiquitinase inhibitor PR-619

**Figure 6.**
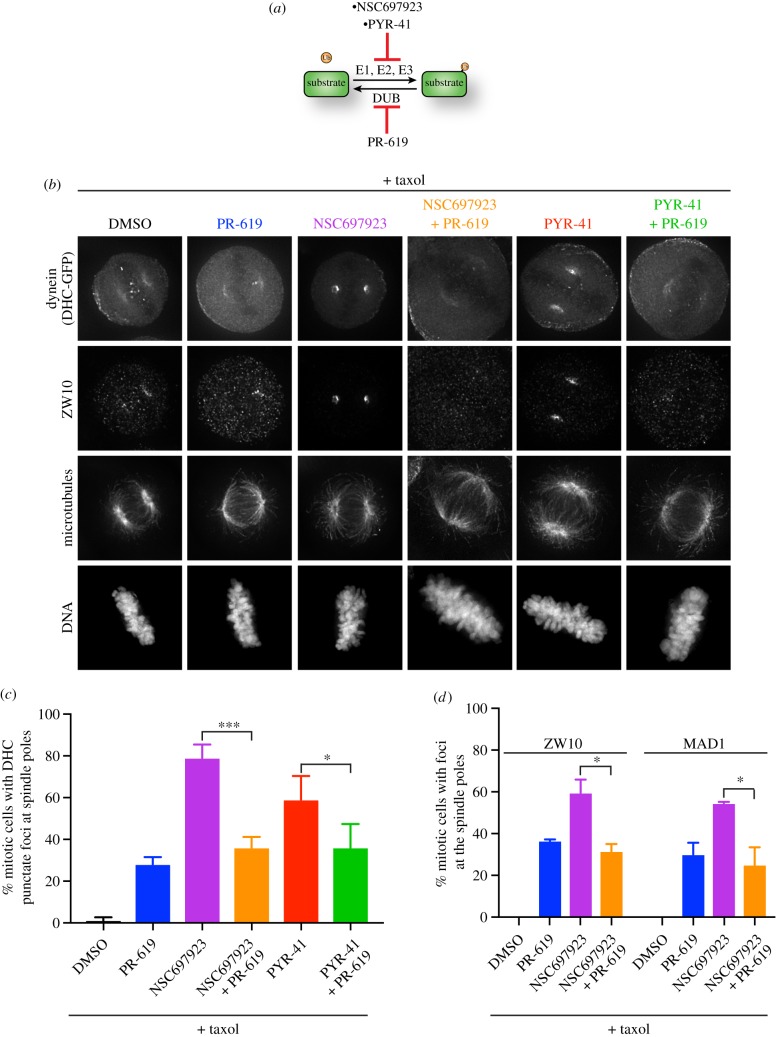
PR-619 suppresses the relocalization of dynein induced by NSC697923 and PYR-41. (*a*) Cartoon illustrating the competing actions of the ubiquitinating enzymes with the deubiquitinases. PR-619 is a non-selective deubiquitinase inhibitor. (*b*) Representative immunofluorescence images of stably expressed dynein heavy chain-GFP (DHC-GFP), ZW10, microtubules (DM1A) and DNA (Hoechst) after treating the cells for 15 min with the indicated compounds. Scale bar, 10 µm. (*c*) Quantification of the percentage of mitotic cells with punctate foci of dynein heavy chain on the spindle poles for the indicated conditions. The data represent the replicate mean ± s.d. Each replicate included 100 cells. Three to four replicates were analysed for all conditions. Statistical significance was determined by unpaired two-tailed *t*-tests. ***p ≤ 0.001; *p ≤ 0.05. (*d*) Quantification of the percentage of mitotic cells with either ZW10 or MAD1 on the spindle poles for the indicated conditions. ZW10 and MAD1 were co-stained in the cells analysed here (and distinct from the cells used to quantify DHC relocalization in panel *c* above). We note that all cells with spindle pole-localized MAD1 also had ZW10 on the spindle poles. A few cells with weak ZW10 signal did not display detectable MAD1 localization. The data represent the replicate mean ± s.d. Each replicate included 100 cells. Two replicates were analysed for each condition. Statistical significance was determined by unpaired two-tailed *t*-tests. *p ≤ 0.05.

If the PYR-41- and NSC697923-mediated effects on dynein localization are due to inhibition of ubiquitination, we reasoned that inhibiting the deubiquitinases might also perturb the localization of dynein due to the dynamic balance of ubiquitin conjugation. To test this premise, we assessed the consequences of treating cells with the non-selective deubiquitinase inhibitor, PR-619 (figure 6*a*). Addition of PR-619 induced dynein accumulation at the spindle poles in a smaller percentage of cells relative to NSC697923 or PYR-41 treatment (figure 6*b*,*c*). However, when PR-619 was simultaneously added to cells with either NSC697923 or PYR-41, the percentage of cells exhibiting accumulated dynein was reduced relative to NSC697923 or PYR-41 treatment alone (figure 6*b*,*c*). Thus, PR-619 treatment appeared to suppress the effects of NSC697923 and PYR-41 on dynein localization. Similar trends were also observed for the relocalization of ZW10 and MAD1 (figure 6*d*). If the effects of these compounds are on-target, these results suggest that ubiquitination dynamics may be critical for controlling dynein localization during mitosis. Inhibiting ubiquitination by NSC697923 or PYR-41 treatment may allow the counteracting deubiquitinases to dominate, thereby resulting in rapid removal of ubiquitin from the substrate and ultimately causing dynein to accumulate at the spindle poles.

Together, our observations that the mitotic localization of dynein can be rapidly altered highlight the highly dynamic nature of dynein regulation within the cell.

## 3. Discussion

As the primary minus-end-directed microtubule motor, dynein is required for numerous cellular processes. Thus, the localization and function of dynein must be precisely regulated. For example, dynamic regulation of dynein localization during mitosis is required for proper positioning of the mitotic spindle within the cell during both metaphase and anaphase [35,36]. Our work highlights that the localization of dynein can be rapidly altered, as we observe a dramatic change in the subcellular distribution of dynein within minutes of drug addition. The ability to quickly retarget dynein to specific sites may allow the cell to dynamically respond to changing needs in force generation or cargo transport.

NSC697923 and PYR-41 could alter dynein localization by several possible mechanisms. First, as dynein is a minus-end-directed motor and the microtubule minus ends are located at the spindle poles, these inhibitors could stimulate dynein motility. In support of this model, dynein relocalization is not observed when the microtubules have been depolymerized (figure 1*c*). A second, non-mutually exclusive mechanism entails an enhanced interaction between dynein and a spindle pole-localized protein due to modification of dynein, a dynein regulator or a spindle-pole protein. This altered interaction could recruit dynein complexes from the cytoplasm, or it could trap dynein complexes that have arrived at the spindle pole after walking along a microtubule. In fact, prior work found that ATP depletion or NDGA treatment prevents the release of dynein from spindle poles and thereby causes a similar accumulation at the poles [23,37–40], albeit with slower kinetics than what we observed here.

Inhibitors targeting both ubiquitin E1 and E2 enzymes cause an increase in dynein signal at spindle poles, suggesting that dynein localization is regulated by ubiquitination. Indeed, another inhibitor of Ubc13, BAY 11–7082 [41], similarly relocalized dynein (data not shown). However, recent *in vitro* studies demonstrated that BAY 11-7082 additionally inhibits the ubiquitin E1 enzyme [42]. As BAY 11-7082 shares structural similarity with NSC697923, it is plausible that dynein relocalization induced by NSC697923 is actually also due to inhibition of the ubiquitin E1 enzyme. Alternatively, our observation that TAK-243, a more potent [30,31] and presumably more specific ubiquitin E1 inhibitor, does not cause the same alteration in dynein localization suggests that there may be another cellular target shared by both NSC697923 and PYR-41. Both PYR-41 and NSC697923 are known to have off-target effects. PYR-41 has been shown to non-specifically cross-link proteins [43] and NSC697923 inhibits the activity of several deubiquitinases *in vitro* [44]. Additionally, it is important to note that TAK-243 treatment does not obviously affect the mitotic spindle on the time scales analysed here. By contrast, PYR-41 treatment causes the spindle poles to move inwards towards the DNA (figure 5*b*). As an inhibitor of the most upstream step in the ubiquitination pathway, PYR-41 should inhibit almost all cellular ubiquitination. Thus, it is particularly surprising that NSC697923 treatment, which should only inhibit the subset of ubiquitination events catalysed by Ubc13, results in potent disassembly of the mitotic spindle (figure 1*b*). Therefore, the distinct microtubule phenotypes we observe after PYR-41 or NSC697923 treatment may be further evidence of off-target effects of one or both compounds.

Regardless of the precise mechanism used, the rapid relocalization of dynein following the addition of NSC697923 and PYR-41 indicates a highly dynamic and precise regulatory network controlling dynein function.

## 4. Experimental procedures

### 4.1. Molecular biology

GFP-tagged constructs for expression in human cells were generated by cloning the human cDNA into a pBABEblast vector containing an N-terminal LAP tag (GFP-TEV-S) as described previously [45].

### 4.2. Cell culture and cell line generation

HeLa cells were cultured in Dulbecco's modified Eagle medium supplemented with 10% fetal bovine serum (GE Healthcare), 100 units ml^−1^ penicillin, 100 units ml^–1^ streptomycin and 2 mM l-glutamine at 37°C with 5% CO_2_. TS20 cells were cultured in Dulbecco's modified Eagle medium supplemented with 10% fetal bovine serum (GE Healthcare), 2 mM l-glutamine and MEM Non-essential amino acids at 35°C with 5% CO_2_.

HeLa cells expressing mouse DHC–GFP were obtained from MitoCheck [46]. HeLa cells expressing GFP-tagged Tctex-type3, ARP1, Lis1, Nde1, CENP-A and CSAP were generated by retroviral infection followed by Blasticidin selection and single-cell sorting. HeLa cells expressing GFP-ZW10 were a gift from Geert Kops (Hubrecht Institute). TS20 cells are a BALB/3T3 cell line containing a temperature-sensitive mutation in the ubiquitin E1 enzyme, Uba1, and were a gift from Nianli Sang (Drexel University). For experiments at the restrictive temperature, TS20 cells were cultured at 39°C for 8 h before treating with PYR-41 for 15 min and then fixing for immunofluorescence.

### 4.3. Chemicals

All chemicals were resuspended in DMSO. NSC697923 (Sigma-Aldrich) was used at 20 µM. Nocodazole (Sigma-Aldrich) was used at 50 nM. MG132 (EMD Biosciences) was used at 10 µM. CC0651 (Thermo Fisher Scientific) was used at 50 µM. Taxol (Life Technologies) was used at 1 µM. PYR-41 (Santa Cruz Biotechnology) was used at 20 µM. TAK-243 (formerly MLN7243) was a gift from Hidde Ploegh (Whitehead Institute) and used at 25 µM. PR-619 (Sigma-Aldrich) was used at 100 µM. ZM447439 (R&D Systems) was used at 2 µM. VX680 (LC Laboratories) was used at 2.5 µM. Flavopiridol (Santa Cruz Biotechnology) was used at 5 µM. AZ3146 (Tocris) was used at 2 µM. BI2536 (Thermo Fisher Scientific) was used at 10 µM. Okadaic acid (VWR) was used at 1 µM. To dose cells, drugs were diluted in fresh media and then added to the cells. For immunofluorescence, cells were dosed for 15 min before fixation.

### 4.4. Immunofluorescence

Cells were plated on glass coverslips coated with poly-l-lysine (Sigma-Aldrich). Cells were fixed with 4% formaldehyde in PHEM buffer (60 mM PIPES, 25 mM HEPES, 10 mM EGTA and 4 mM MgSO_4_, pH 7) for 10 min. Blocking and all antibody dilutions were performed using AbDil (20 mM Tris, 150 mM NaCl, 0.1% Triton X-100, 3% BSA and 0.1% NaN_3_, pH 7.5). PBS plus 0.1% Triton X-100 (PBS-TX) was used for washes. GFP-Booster (Chromotek; 1 : 200 dilution) was used to amplify the fluorescence of the GFP-tagged transgenes. DM1A was used to stain the microtubules (Sigma-Aldrich; 1 : 3000 dilution). For staining of NuMA, ab36999 (Abcam) was used at a 1 : 500 dilution. For staining of Spindly, rabbit anti-Spindly [47] (a gift from Erich Nigg and Anna Santamaria, Max Planck Institute of Biochemistry) was used at a 1 : 1000 dilution. For staining of MAD1, ab5783 (Abcam) was used at a 1 : 1000 dilution. For staining of MAD2, A300-301A (Bethyl Laboratories) was used at 1 µg ml^−1^. For staining of Bub1, ab54893 (Abcam) was used at a 1 : 500 dilution. For staining of ZW10, ab21582 (Abcam) was used at 1 µg ml^−1^. Cy3- and Cy5-conjugated secondary antibodies (Jackson ImmunoResearch Laboratories) were used at a 1 : 300 dilution. DNA was visualized by incubating cells in 1 µg ml^−1^ Hoechst-33342 (Sigma-Aldrich) in PBS-TX for 10 min. Coverslips were mounted using PPDM (0.5% *p*-phenylenediamine and 20 mM Tris-Cl, pH 8.8, in 90% glycerol).

### 4.5. Fluorescence microscopy

Images were acquired on a DeltaVision Core microscope (Applied Precision) equipped with a CoolSnap HQ2 CCD camera (Photometrics). A 100×, 1.4 NA U-PlanApo objective (Olympus) was used to image fixed cells, and a 60×, 1.42 NA Plan Apo N objective (Olympus) was used for live-cell imaging. Images were deconvolved and maximally projected. The fluorescence is not scaled equivalently in each panel to clearly demonstrate the qualitative localization of each protein.

To quantify the spindle pole accumulation frequency of dynein heavy chain, each replicate included 100 cells for each condition. The percentage of mitotic cells with clear, strong foci of the dynein-GFP signal on the spindle poles was denoted. 3-4 biological replicates were analysed for each condition and the mean percentage of cells (±s.d.) with strong dynein signal for those replicates was plotted. For ZW10 and MAD1, 2 biological replicates were analysed for each condition and the mean percentage of cells (±s.d.) with spindle pole-localized signal for those replicates was plotted.

Line scans were generated through the ‘Plot profile’ function in ImageJ, using maximally projected, but not deconvolved, images.

## Supplementary Material

Figure S1. NSC697923 induces accumulation of dynein at the mitotic spindle poles without affecting NuMA.

## Data accessibility

This article has no additional data.
